# Prevalence of sexual dysfunction and its association with psychological symptoms in drug-naive major depressive disorder patients in West China

**DOI:** 10.3389/fpsyt.2023.1291988

**Published:** 2023-12-07

**Authors:** Fugui Jiang, Zenghui Liu, Xianglong Wu, Arui Tan, Xiaohong Qin, Rong Cheng Su, Hui Li, Huan Wang, Jun Xiao, Bo Zhou

**Affiliations:** ^1^Sichuan Provincial Center for Mental Health, Sichuan Academy of Medical Sciences & Sichuan Provincial People's Hospital, Chengdu, China; ^2^Key Laboratory of Psychosomatic Medicine, Chinese Academy of Medical Sciences, Chengdu, China

**Keywords:** major depressive disorder, sexual dysfunction, ASEX, prevalence, psychological symptom

## Abstract

**Background:**

Sexual dysfunction is commonly observed in individuals with Major Depressive Disorder (MDD), along with various psychological symptoms such as anxiety, somatic complaints, interpersonal sensitivity, and obsessive-compulsive tendencies. However, there is a research gap in understanding the impact of these psychological symptoms on sexual functioning in MDD. Furthermore, there is limited data on the incidence of sexual dysfunction among drug-naive MDD patients in West China. This study aims to determine the prevalence of sexual dysfunction in this patient population and explore its association with other psychological indicators.

**Methods:**

We conducted a retrospective analysis of patient data from October 2020 to September 2022 using propensity score matching. A focused group of 165 males and 490 females was selected from a total of 1941 MDD patients. This allowed for a comparative analysis of demographic data, as well as scores from the Self-Rating Depression Scale (SDS), Self-Rating Anxiety Scale (SAS), and Symptom Checklist-90 (SCL-90), the Arizona Sexual Experience Scale (ASEX).

**Results:**

Our findings reveal that 46.2% of drug-naive MDD patients experienced sexual dysfunction. Notably, there was a higher prevalence of sexual dysfunction among female patients (50.3%) compared to males (37.5%). MDD patients without sexual dysfunction consistently exhibited higher SDS scores than those with sexual dysfunction (*p* < 0.01), There were no statistically significant differences between male and female MDD patients with or without concomitant sexual dysfunction in terms of Somatic complaints, Obsessive-compulsive, Interpersonal sensitivity, Anxiety, Phobic anxiety, Paranoid ideation, Psychoticism and Diet/sleep difficulties (*p* > 0.05). In addition, male MDD patients with sexual dysfunction showed a emerging trend towards elevated Hostility scores on the SCL-90 (*p* = 0.058), male MDD patients with sexual dysfunction showed an increasing trend in hostility scores on the SCL-90, whereas female MDD patients with sexual dysfunction did not show such a trend.

**Conclusion:**

The study highlights a significant gender difference in the prevalence of sexual dysfunction among MDD patients, with females being more susceptible than males. There is a positive correlation between the severity of depression and sexual dysfunction in both genders. Interestingly, male MDD patients demonstrated a potential protective effect of hostility against sexual dysfunction, which was not observed in female patients.

## 1 Introduction

Major Depressive Disorder (MDD) is a widespread mental illness observed worldwide. In the United States, the prevalence of MDD is 10.4% for a duration of 12 months and 20.6% over a lifetime (1). In China, MDD takes precedence as the primary mood disorder, with a lifetime prevalence of 3.4% and a 12-month prevalence of 2.1% (2). The impact of the COVID-19 pandemic has further exacerbated this situation, resulting in a significant increase in depression rates (3, 4). Among mental health conditions, MDD stands out as a key contributor to mortality and disability in the general population. When measured in terms of “years lived with disability,” MDD ranks second as the leading cause of chronic disease burden (5). Moreover, MDD is associated with a higher risk of chronic medical ailments such as diabetes, heart disease, and stroke (6), amplifying its overall impact on disease burden. Tragically, MDD can lead to suicide, with individuals suffering from depression being approximately 20 times more likely to die by suicide compared to the general population (7).

Sexual dysfunction impacts around 43% of females and 31% of males (8) and is a significant comorbidity for depressive symptoms. Its identification as a sign of major depressive disorder (MDD) is apparent in standardized forms for rating depression that include a question evaluating the loss of interest in sexual activities. The connection between sexual dysfunction and depression is cyclical; the former raises the risk for the latter by 2.3 to 3.1-fold (9). 25 to 75% of individuals with depression experience sexual dysfunction, with the intensity of symptoms corresponding to the severity of the depression (10). Treatments for depression, particularly selective serotonin reuptake inhibitors (SSRIs), are linked to a 70% rise in the occurrence of treatment-associated sexual dysfunction (11). Although the usage of antidepressants may influence studies on sexual function (12, 13), many sexual symptoms decrease as mental well-being improves. Various researchers have evaluated the prevalence of sexual dysfunction in patients with MDD (14–16), but there is a scarcity of empirical research, particularly in Western China, on individuals with untreated MDD.

Depression often occurs together with various psychological factors, which can also affect reported sexual problems. These psychological factors include physical symptoms, worry, aggression, and changes in eating or sleeping behaviors, among other things (17–20). While numerous studies have focused on depression, research on depression coexisting with psychological symptoms is still in its early stages. It is crucial to distinguish and explore the various complex psychological aspects that could potentially impact sexual dysfunction. The connection between sexual dysfunction and demographic characteristics, such as age and education (7, 21), further complicates the study of MDD patients with concurrent sexual dysfunction. Given the intricate relationship between sexual dysfunction and MDD in patients. The objective of this study is to determine the prevalence of sexual dysfunction among drug-naive patients with Major Depressive Disorder (MDD) in West China and to explore its association with various psychological symptoms through the use of propensity score matching. These symptoms include, but are not limited to, anxiety, somatic complaints, interpersonal sensitivity, and obsessive-compulsive tendencies. The study aims to fill the research gap in understanding the impact of these psychological factors on sexual functioning in MDD patients and contribute to the limited data on this subject in the context of West China.

## 2 Methods

### 2.1 Participants and study design

The design of this study is a retrospective cohort conducted at a single center. The Ethics Committee of Sichuan Provincial People’s Hospital approved the study in 2020, and the ethics committees of all participating entities provided further endorsement (approval number: Res-2020-290). To ensure participant confidentiality, all identifying details such as names were anonymized. Table 1 presents the data from October 2020 to September 2022, where the Sichuan Provincial Center for Mental Health attended to a total of 1,941 individuals with Major Depressive Disorder (MDD). Inclusion criteria for this study were as follows: (1) Meeting the diagnostic criteria for MDD outlined in the Diagnostic and Statistical Manual of Mental Disorders, Fifth Edition, and confirmation of diagnosis by two experienced psychiatrists. (2) No presence of significant physical illness, bipolar affective disorder with depressive episodes, other psychiatric disorders, or immune or endocrine system-related diseases. (3) Absence of recent infections or vaccinations. Participants were excluded if they: (1) Demonstrated depressive symptoms associated with physical health issues or other psychiatric disorders. (2) Had severe physical conditions. (3) Had a history of other psychiatric disorders and substance abuse. (4) Were currently taking antidepressant medications or drugs that enhance sexual performance.

**Table 1:** Demographic and baseline data of all MDD participants (M ± S/n [%]).

	MDD with SD (*n* = 700.00)	MDD without SD (*n* = 816)	*p*	MDD with SD (*n* = 670.00)	MDD without SD (*n* = 670)	*p*
Gender (n)			<0.001			0.76
Male	180 (25.70%)	300 (36.80%)		185 (27.60%)	180 (26.90%)	
Female	520 (74.30%)	516 (63.20%)		485 (72.40%)	490 (73.10%)	
Age (years)	41.83 ± 18.20	46.15 ± 19.00	<0.010	45.30 ± 18.89	44.67 ± 18.18	0.54
Race			0.917			0.64
Han	659 (94.00%)	766 (93.90%)		633 (94.50%)	629 (93.90%)	
others	42 (6.00%)	50 (6.10%)		37(5.50%)	41.00 (6.10%)	
Marital status			0.070			0.89
Single	176 (25.10%)	252 (30.90%)		169 (25.20%)	174 (26.00%)	
Married/partnered	478 (68.10%)	516 (63.20%)		460 (68.70%)	450 (67.20%)	
Divorced/separated	26 (3.70%)	31 (3.80%)		25 (3.70%)	26 (3.90%)	
Widowed	21 (3.00%)	17 (2.10%)		16 (2.40%)	20 (3.00%)	
Education, year	12.27 ± 3.16	12.46 ± 4.34	0.321	12.31 ± 3.39	12.24 ± 3.16	0.68
SAS	58.48 ± 11.33	57.78 ± 13.58	0.272	57.89 ± 13.56	58.39 ± 11.26	0.47
SDS	65.35 ± 8.82	67.84 ± 9.95	<0.010	68.04 ± 10.00	64.63 ± 8.37	<0.01
SCL-90						
Somatic complaints	2.31 ± 0.81	2.29 ± 0.88	0.656	2.29 ± 0.88	2.32 ± 0.80	0.49
Obsessive-compulsive	2.72 ± 0.80	2.69 ± 0.93	0.496	2.71 ± 0.93	2.69 ± 0.79	0.75
Interpersonal sensitivity	2.44 ± 0.90	2.41 ± 1.03	0.557	2.43 ± 1.03	2.38 ± 0.87	0.32
Depression	2.78 ± 0.86	2.77 ± 1.08	0.926	2.79 ± 1.08	2.74 ± 0.84	0.31
Anxiety	2.61 ± 0.89	2.51 ± 1.03	0.050	2.53 ± 1.03	2.59 ± 0.87	0.21
Hostility	2.30 ± 0.91	2.19 ± 0.99	0.017	2.20 ± 0.99	2.21 ± 0.88	0.88
Phobic anxiety	2.14 ± 0.87	2.16 ± 0.99	0.763	2.17 ± 0.99	2.13 ± 0.87	0.52
Paranoid ideation	2.18 ± 0.91	2.12 ± 0.98	0.232	2.14 ± 0.97	2.11 ± 0.88	0.68
Psychoticism	2.25 ± 0.77	2.22 ± 0.91	0.407	2.23 ± 0.91	2.21 ± 0.74	0.60
Diet/sleep difficulties	2.62 ± 0.80	2.65 ± 0.90	0.538	2.66 ± 0.89	2.68 ± 0.74	0.73
Mean score	2.47 ± 0.71	2.43 ± 0.87	0.411	2.45 ± 0.87	2.44 ± 0.70	0.85
Total score	221.89 ± 64.34	218.89 ± 77.95	0.412	220.10 ± 77.87	219.36 ± 62.88	0.85

### 2.2 Source of data

We gathered baseline demographic and clinical information. We assessed the severity of depression and anxiety using the Self-Depression Scale (SDS) and the Self-Rating Anxiety Scale (SAS) respectively. Psychological symptoms were evaluated using the Symptom Checklist-90 (SCL-90), and sexual dysfunction (SD) was screened using the Arizona Sexual Experience Scale (ASEX). Self-Rating Anxiety Scale (SAS): The SAS measures levels of anxiety and mood over the previous week with 20 items divided into four categories. An overall score of 20, multiplied by 1.25, reflects the severity of anxiety. This tool has good internal consistency with a Cronbach’s alpha of 0.821 (22). Self-Depression Scale (SDS): We used the SDS to assess the degree of depression in each participant. It consists of 20 items that are rated on a scale from 1 to 4, with higher scores indicating more frequent symptoms. Higher scores suggest more severe depressive symptoms (23). Symptom Checklist-90 (SCL-90): This questionnaire comprises ten factors, with each item graded based on the severity of symptoms. A total score above 160 points or factor scores of ≥2 indicate significant symptoms, with higher scores suggesting severe psychiatric issues (24). Arizona Sexual Experience Scale (ASEX): This scale assesses five domains of sexual function, categorizing SD based on specific criteria. We chose the Chinese ASEX version due to its effectiveness in Chinese individuals with Major Depressive Disorder (MDD), and it has a Cronbach’s alpha of 0.85 (25).Sexual dysfunction is defined as (i) a total ASEX score of 19 or above, (ii) any item with a score of 5 or above, or (iii) any three items with a score of 4 or above. The ASEX is a reliable, valid, and sensitive tool to measure SD in both males and females. All participants received guidance from a psychiatrist before completing the scale.

### 2.3 Methods for statistical analysis

The statistical analysis of the study results was conducted using SPSS 27.0. All study outcomes were presented using mean ± standard deviations or n (%). In order to accurately compare the different groups (MDD without SD vs. MDD with SD), both qualitative and quantitative variables were analyzed using χ2 or independent sample t-tests, respectively. To minimize any influences from confounding factors and systematic biases, the statistical analyses incorporated Propensity Score Matching (PSM). By utilizing PSM, the baseline characteristics of the MDD groups (with and without SD) were balanced. Binary logistic regression was employed to identify factors contributing to the incidence of MDD with SD. Statistical significance was considered when the *p*-value was less than 0.05.

## 3 Results

### 3.1 Description of overall sample

Out of the initial 1,941 patients that were approached, a total of 1,516 participants were suitable for analysis after excluding 425 patients for various reasons. These reasons included physical illnesses (*n* = 176), depressive symptoms caused by external factors like stroke, medication, or pregnancy (*n* = 153), a history of other mental disorders (*n* = 54), and missing data (*n* = 42). The final group for this study consisted of 1,036 females and 480 males, with an average age of 43.8 ± 18.7 years (age range from 18 to 88 years). Of these MDD patients, a predominant 93.9% (*n* = 1,424) were of Han Chinese ethnicity, while the remaining 6.1% (*n* = 92) belonged to various other ethnic groups. Regarding marital status, the majority of our participants were married, accounting for 65.5% (*n* = 993), followed by 28.2% (*n* = 428) who were unmarried, 3.8% (*n* = 57) divorced, and 2.5% (*n* = 38) widowed. The average educational attainment among the participants was 12.36 years, with a standard deviation of 3.75 years, indicating a relatively uniform educational background across the study sample. Among the total of 1,516 MDD patients, 46.2% (700 cases) were diagnosed with concurrent sexual dysfunction (SD) (Table 1).

### 3.2 Sexual dysfunction in males and females with MDD

Low sex drive was reported in both males and females, (31.9% vs.37.8%, *p* < 0.01) and difficulties in sexual arousal (27.1% vs.30.3%, *p* = 0.03). Within the male MDD patient population, 31.9% experienced issues with penile erection, while 30.7% of female patients faced challenges with vaginal lubrication (*p* = 0.21). Furthermore, males and females reported difficulties in achieving orgasm (26.0% vs. 28.0%, *p* = 0.20) and unsatisfying orgasms (25.8%vs.26.4%, *p* = 0.04). In regards to the occurrence of total sexual dysfunction, it was found that females exhibited a significantly higher frequency compared to males (50.1% vs. 37.5%, *p* < 0.01). Evaluated with the item scores (Positive items ≥4), Low sex drive and penile erection are the most prevalent sexual dysfunctions among male MDD patients, whereas female MDD patients primarily experience low sex drive as their most common sexual dysfunction (Table 2).

**Table 2:** Sexual dysfunction in male and female MDD patients.

ASEX (Positive items≥4), n (%)	Male (480)	Female (1036)	χ^2^	*p*-value
Quantify sex drive	153 (31.9%)	392 (37.8%)	8.80	<0.01
Arousal	130 (27.1%)	314 (30.3%)	4.65	0.03
Vaginal lubrication or penile erection	153 (31.9%)	318 (30.7%)	1.55	0.21
Ability to reach orgasm	125 (26.0%)	290 (28.0%)	1.67	0.20
Satisfaction from orgasm	27 (25.8%)	273 (26.4%)	4.23	0.04
Total Sexual dysfunction	180 (37.5%)	520 (50.1%)	21.264	<0.01

ASEX, Arizona Sexual Experience Scale; Total Sexual dysfunction was defined as (i) a total score ≥ 19, (ii) ≥ 5 in any item, or (iii) ≥ 4 in three items.

To perform the Propensity Score Matching (PSM) analysis, predictive variables including age, marital status, race, and education were selected. A matching tolerance of 0.1 was established. Tables 2^3^ present the results after PSM. The primary objective of this research was to determine the differences in psychological factors between MDD patients with and without SD. By balancing demographic variables through the PSM procedure, a comparison of the main and secondary results could be made under similar conditions. Out of the 480 male MDD patients, 180 experienced SD, giving them a prevalence rate of 37.5%. Similarly, 520 out of the 1,036 female MDD patients displayed signs of SD, resulting in a detection rate of 50.3%. After PSM, no significant differences were found in terms of age, marital status, race, and education between the groups. Additionally, both male and female MDD patients with SD exhibited significantly higher SDS scores compared to those without SD (*p* < 0.01). There were no statistically significant differences between male and female MDD patients with or without concomitant sexual dysfunction in terms of Somatic complaints, Obsessive-compulsive, Interpersonal sensitivity, Anxiety, Phobic anxiety, Paranoid ideation, Psychoticism and Diet/sleep difficulties (*p* > 0.05),Male MDD patients without SD had slightly higher Hostility scores on the SCL-90 (*p* = 0.058) and a slight decrease in Diet/sleep difficulties scores on the SCL-90 (*p* = 0.057) compared to those with SD. However, these trends were not observed among female MDD patients (Tables 3 4).

**Table 3:** Comparison of male MDD participants before and after propensity score matching.

Before matching				After matching		
	MDD without SD (*n* = 300)	MDD with SD (*n* = 180)	*p*	MDD without SD (*n* = 165)	MDD with SD (*n* = 165)	*p*
Age (years)			<0.010			0.51
18.00–30.00	99 (33.00%)	41 (22.80%)		38 (23.00%)	41 (24.80%)	
30.00–45.00	76 (25.30%)	28 (15.60%)		38 (23.00%)	28 (17.00%)	
45.00–60.00	75 (25.00%)	50 (27.80%)		42 (25.50%)	50 (30.30%)	
≥60.00	50 (16.70%)	61 (33.90%)		47 (28.50%)	46 (17.90%)	
Race			0.257			0.83
Han	283 (94.30%)	165 (91.70%)		153 (92.70%)	152 (92.10%)	
others	17 (5.70%)	15 (8.30%)		12 (7.30%)	13 (7.90%)	
Marital status			0.084			0.36
Single	100 (%)	41 (22.80%)		39 (23.60%)	41 (24.80%)	
Married	188 (%)	132 (73.30%)		120 (72.70%)	118 (71.50%)	
Divorced	9 (%)	6 (3.30%)		4 (2.40%)	6 (3.60%)	
Widowed	3 (%)	1 (0.60%)		2 (1.20%)	0 (0%)	
Education, year	12.19 ± 3.22	12.27 ± 3.25	0.779	12.03 ± 3.28	12.34 ± 3.22	0.39
SAS	56.43 ± 11.44	55.63 ± 13.31	0.483	55.03 ± 11.07	55.99 ± 13.49	0.48
SDS	64.50 ± 8.62	67.72 ± 9.84	<0.010	62.22 ± 7.31	67.63 ± 9.80	<0.01
SCL-90.00						
Somatic complaints	2.15 ± 0.78	2.13 ± 0.90	0.733	2.07 ± 0.73	2.14 ± 0.92	0.43
Obsessive-compulsive	2.62 ± 0.82	2.62 ± 1.01	0.967	2.52 ± 0.80	2.66 ± 1.03	0.18
Interpersonal sensitivity	2.39 ± 0.92	2.25 ± 1.02	0.124	2.24 ± 0.84	2.29 ± 1.03	0.65
Depression	2.66 ± 0.86	2.67 ± 1.15	0.937	2.54 ± 0.81	2.73 ± 1.16	0.10
Anxiety	2.48 ± 0.90	2.39 ± 1.04	0.324	2.40 ± 0.85	2.43 ± 1.06	0.78
Hostility	2.35 ± 0.91	2.06 ± 0.90	0.001	2.27 ± 0.88	2.09 ± 0.92	0.06
Phobic anxiety	1.99 ± 0.82	2.03 ± 0.99	0.619	1.91 ± 0.75	2.06 ± 1.01	0.11
Paranoid ideation	2.13 ± 0.93	2.08 ± 1.01	0.578	1.97 ± 0.84	2.12 ± 1.03	0.13
Psychoticism	2.23 ± 0.80	2.15 ± 0.95	0.286	2.12 ± 0.72	2.18 ± 0.97	0.54
Diet/sleep difficulties	2.43 ± 0.84	2.55 ± 0.96	0.166	2.40 ± 0.82	2.59 ± 0.98	0.06
Mean score	2.37 ± 0.71	2.32 ± 0.90	0.510	2.27 ± 0.65	2.36 ± 0.92	0.31
Total score	213.41 ± 64.10	208.99 ± 81.13	0.509	204.32 ± 58.26	212.28 ± 82.48	0.31

**Table 4:** Comparison of female MDD participants before and after propensity score matching.

Before matching				After matching		
	MDD without SD (*n* = 516)	MDD with SD (*n* = 520)	*p*	MDD without SD (*n* = 490)	MDD with SD (*n* = 490)	*p*
Age (years)			0.104			0.69
18.00–30.00	166 (32.20%)	148 (28.40%)		147 (30.00%)	148 (30.20%)	
30.00–45.00	110 (21.30%)	92 (17.90%)		104 (21.20%)	91 (18.60%)	
45.00–60.00	143 (27.70%)	157 (30.10%)		142 (29.00%)	143 (29.20%)	
≥60.00	97 (18.80%)	123 (23.60%)		97 (19.80%)	108 (22.00%)	
Race			0.403			0.89
Han	483 (93.60%)	493 (94.80%)		464 (94.70%)	463 (94.50%)	
others	33 (6.40%)	27 (5.20%)		26 (5.30%)	27 (5.50%)	
Marital status			0.485			0.72
Single	152 (29.50%)	136 (26.10%)		134 (%)	136 (%)	
Married	328 (63.60%)	344 (66.20%)		320 (%)	315 (%)	
Divorced	22 (4.30%)	20(3.80%)		22 (%)	19 (%)	
Widowed	14 (2.70%)	20(3.80%)		14 (%)	20 (%)	
Education, year	12.32 ± 3.13	12.51 ± 4.66	0.426	12.34 ± 3.13	12.30 ± 3.45	0.84
SAS	59.68 ± 11.10	58.51 ± 13.60	0.131	59.59 ± 11.17	58.62 ± 13.77	0.23
SDS	65.85 ± 8.90	67.90 ± 10.00	0.001	65.66 ± 8.79	67.93 ± 9.86	<0.01
SCL-90.00						
Somatic complaints	2.40 ± 0.81	2.34 ± 0.86	0.295	2.39 ± 0.82	2.36 ± 0.86	0.50
Obsessive-compulsive	2.78 ± 0.78	2.72 ± 0.90	0.231	2.76 ± 0.78	2.74 ± 0.90	0.64
Interpersonal sensitivity	2.47 ± 0.89	2.47 ± 1.03	0.981	2.43 ± 0.88	2.49 ± 1.03	0.33
Depression	2.84 ± 0.86	2.81 ± 1.06	0.543	2.82 ± 0.85	2.83 ± 1.06	0.87
Anxiety	2.69 ± 0.88	2.56 ± 1.02	0.026	2.67 ± 0.88	2.58 ± 1.02	0.13
Hostility	2.27 ± 0.91	2.23 ± 1.01	0.496	2.25 ± 1.02	2.24 ± 0.90	0.88
Phobic anxiety	2.23 ± 0.89	2.20 ± 0.99	0.594	2.21 ± 0.89	2.21 ± 0.99	0.93
Paranoid ideation	2.20 ± 0.90	2.13 ± 0.96	0.215	2.17 ± 0.88	2.15 ± 0.97	0.72
Psychoticism	2.26 ± 0.75	2.24 ± 0.89	0.645	2.24 ± 0.75	2.26 ± 0.89	0.75
Diet/sleep difficulties	2.73 ± 0.75	2.68 ± 0.87	0.325	2.72 ± 0.75	2.69 ± 0.88	0.53
Mean score	2.52 ± 0.71	2.47 ± 0.85	0.297	2.50 ± 0.71	2.49 ± 0.85	0.81
Total score	226.82 ± 64.02	222.26 ± 76.53	0.298	225.03 ± 63.99	223.98 ± 76.45	0.82

**Figure 1:**
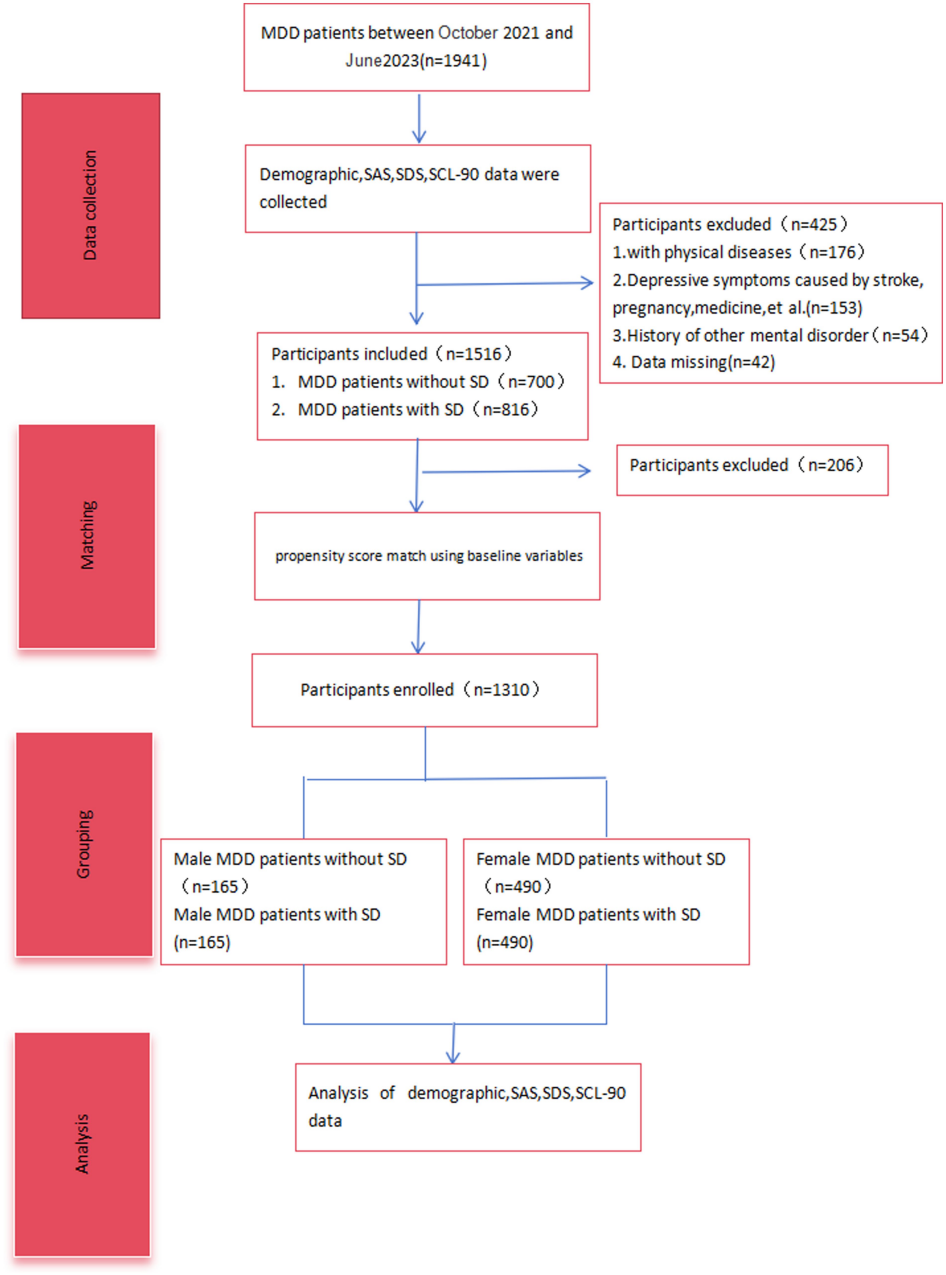
Flowchart.

Factors that Increase the Risk of MDD with SDTo check for plagiarism, a binary logistic regression analysis was conducted where the dependent variable was the presence or absence of SD in individuals with MDD. The variables considered as independent were SAS, SDS, and SCL-90 scores. The analysis demonstrated that the occurrence of SD in both male (OR = 1.084, *p* < 0.01) and female (OR = 1.026, p < 0.01) MDD patients could be independently predicted by the SDS score, as displayed in Figure 1.

## 4 Discussion

**Figure 2:**
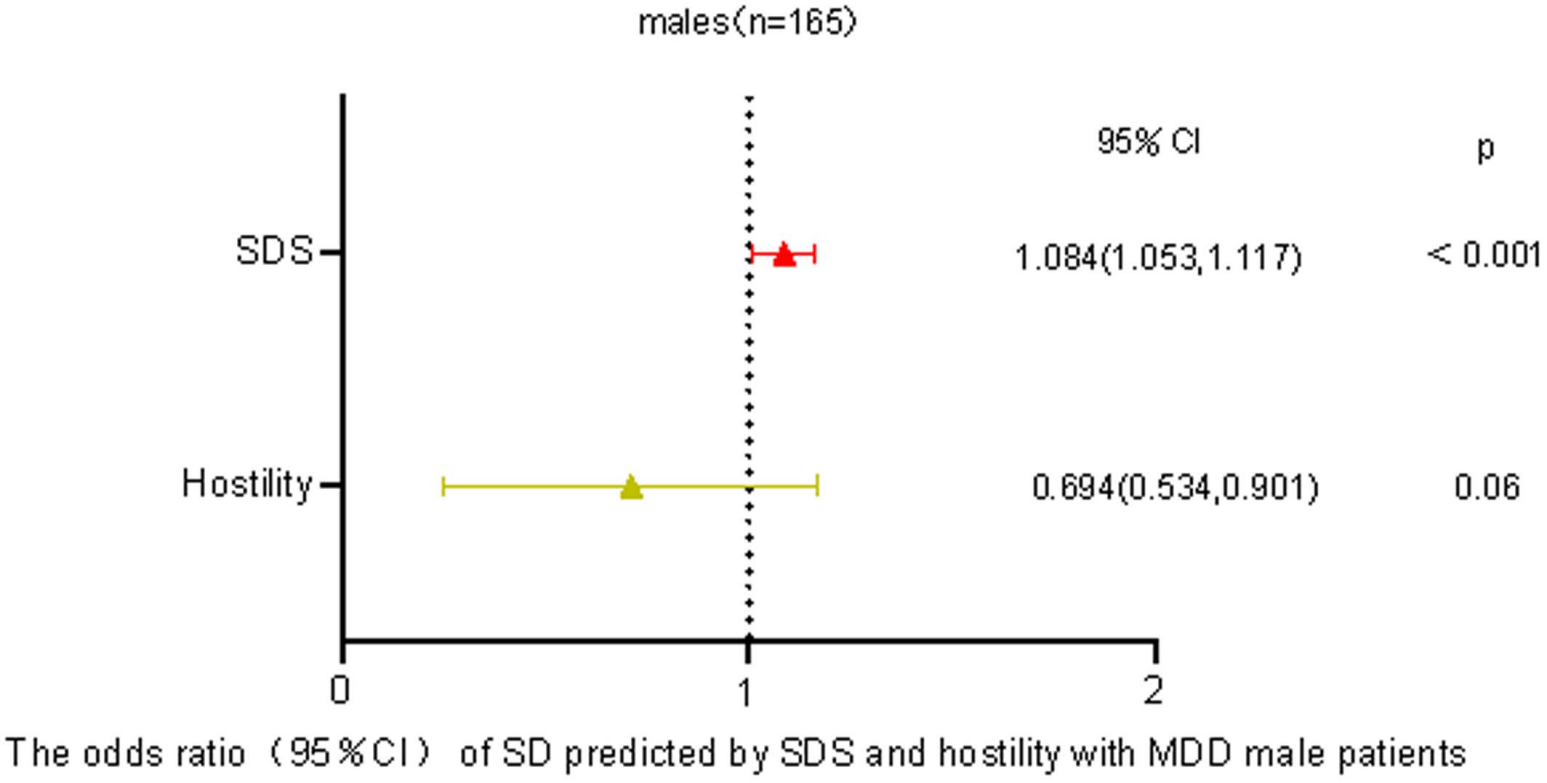
Predicting odds of sexual dysfunction by self-reported depression and hostility in male patients with MDD.

Our investigation into individuals diagnosed with Major Depressive Disorder (MDD) revealed a notable disparity between genders when it came to sexual dysfunction. Female patients reported a significantly higher prevalence compared to their male counterparts. This observation suggests a connection between the severity of depression and the occurrence of sexual dysfunction across genders. Interestingly, our findings indicate that hostility may act as a protective factor against sexual dysfunction among males with MDD, while this relationship was not observed in females (see Figures 2 3).

**Figure 3:**
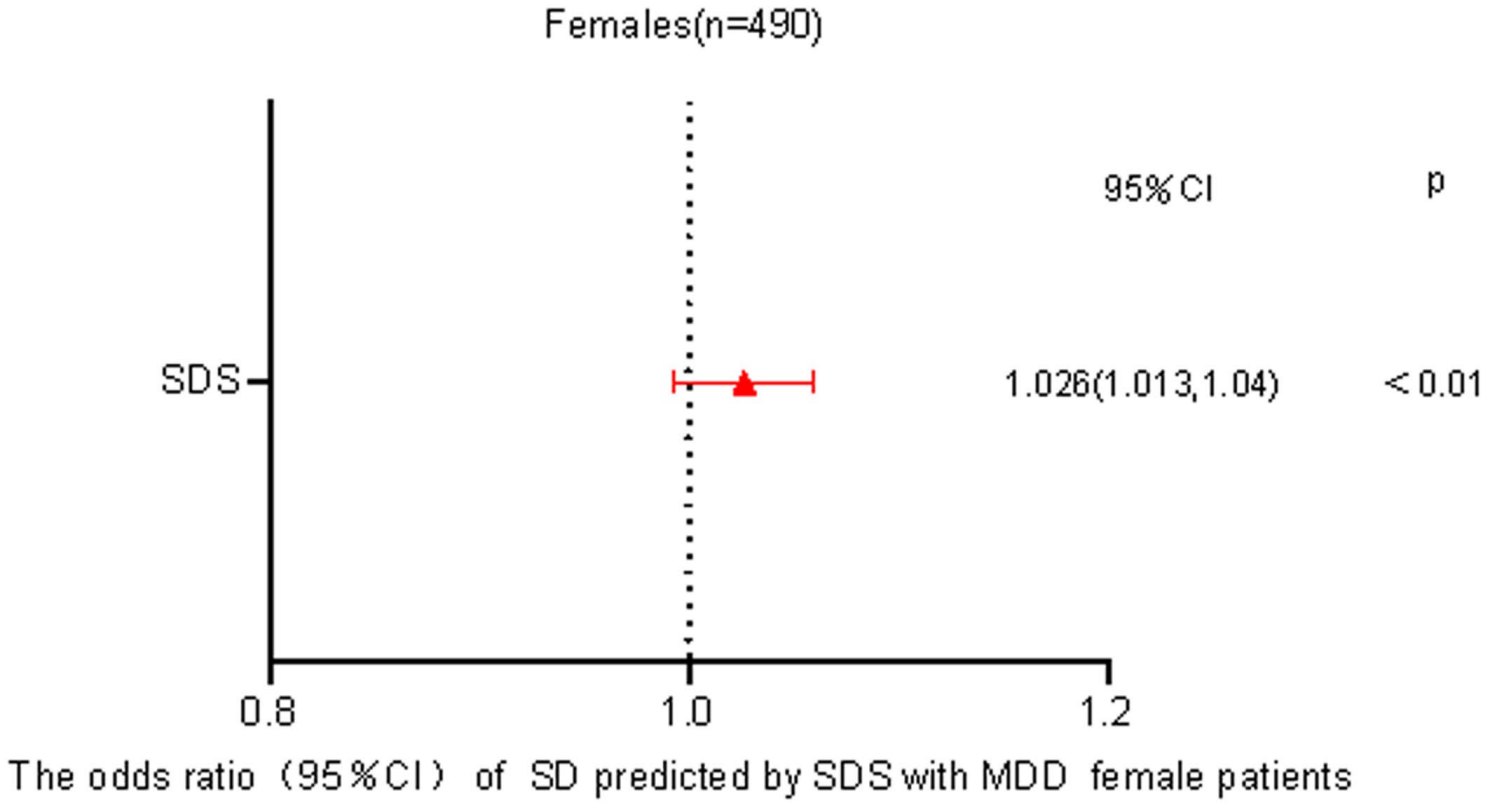
Predicting odds of sexual dysfunction by self-reported depression in female patients with MDD.

A comprehensive analysis comprising 12 studies investigating depressive disorders revealed that 82.75% of females and 63.26% of males encountered sexual problems, excluding those receiving medication (14). Another investigation on sexual issues in Asia disclosed that 37% of males and 45% of females in China undergo at least one form of sexual dysfunction. Generally, within the broader population, females consistently report a higher prevalence of sexual concerns compared to males (26, 27). In our assessment, 46.2% of patients diagnosed with major depressive disorder acknowledged experiencing sexual dysfunction, with a significantly higher occurrence in females (50.3%) as compared to males (37.5%). This finding is consistent with previous studies, which have indicated a more pronounced sexual dysfunction in females with MDD (28). One suggested explanation for this gender discrepancy centers around variations in the neurobiological aspect. Depressed females, in contrast to their mentally healthy counterparts, display reduced activity in specific regions of the brain, including the hypothalamus, septal region, anterior cingulate gyrus, and parahippocampal gyrus (29). However, recent years have observed an increased focus on men’s sexual health, although discussions relating to sexuality are still regarded as somewhat taboo in many Asian cultures (30). A prevalent lack of sexual knowledge often leads to dysfunction, particularly among women (31).

Our research supports the claim that the severity of depression in MDD patients is connected to the prevalence of sexual dysfunction, aligning with previous studies (10). Sexual dysfunction tends to worsen with increasing severity of depression (32). The association between sexual dysfunction prevalence and depression severity in MDD patients was not found with the SCL-90 metrics, possibly because the SCL-90 focuses mainly on depression and does not cover the entire range of clinical symptoms. It is important to note that various factors, such as gender differences, testosterone levels, brain structure, medication effects, and coexisting conditions like alcohol dependence, can influence the relationship between sexual dysfunction prevalence and depression severity in MDD patients (33–36). Another significant finding in our study relates to the potential protective effect of higher hostility levels (measured by the SCL-90) against sexual dysfunction (37, 38). Patients with pronounced hostility levels appeared to be less susceptible to sexual dysfunction, which differs from previous research. One possible explanation is that heightened hostility may be a result of factors like increased testosterone levels, which could enhance sexual function while also increasing feelings of hostility (34). Additionally, meta-analyses have emphasized the impact of different personality traits on various aspects of human sexuality (39–41). It should be noted that our study primarily focused on patients at high risk of suicide or aggression. Such individuals, characterized by heightened hostility and aggression, may inherently exhibit increased sexual behaviors (42).

Yet, there are constraints to our study. It was a clinical study conducted retrospectively in a single-center and small number of men, neglecting the consideration of potential economic factors’ impact on sexual dysfunction, which could potentially introduce confounding variables. To obtain a more exhaustive analysis and enhance the generalizability of our findings, a more comprehensive evaluation of clinical particulars would have been beneficial. Lastly, our research did not incorporate a sample size customized to its specific objectives.

## 5 Conclusion

Our study confirms a significant gender disparity in sexual dysfunction among individuals diagnosed with Major Depressive Disorder (MDD), with females reporting a higher prevalence than males. These findings align with previous research highlighting the consistent reporting of heightened sexual concerns among females. Our research also underscores the potential link between depression severity in MDD patients and the prevalence of sexual dysfunction. A remarkable observation from our study is the potential protective role of hostility against sexual dysfunction in male MDD patients.

## Data availability statement

The original contributions presented in the study are included in the article/supplementary material, further inquiries can be directed to the corresponding authors.

## Ethics statement

The studies involving humans were approved by the Ethics Committee of Sichuan Provincial People's Hospital, Sichuan Academy of Medical Sciences & Sichuan Provincial People's Hospital. The studies were conducted in accordance with the local legislation and institutional requirements. The ethics committee/institutional review board waived the requirement of written informed consent for participation from the participants or the participants' legal guardians/next of kin because the study was conducted in accordance with the principles of the Declaration of Helsinki. Because of the retrospective nature of the study, patient consent for inclusion was waived.

## Author contributions

FJ: Conceptualization, Methodology, Resources, Writing – original draft, Writing – review & editing. ZL: Data curation, Formal analysis, Writing – review & editing. XW: Data curation, Project administration, Software, Writing – review & editing. AT: Formal analysis, Investigation, Writing – review & editing. XQ: Investigation, Supervision, Validation, Visualization, Writing – original draft. RS: Methodology, Writing – review & editing. HL: Funding acquisition, Project administration, Resources, Writing – review & editing. HW: Resources, Software, Writing – original draft. JX: Resources, Funding acquisition, Project administration, Supervision, Validation, Visualization, Writing – review & editing. BZ: Funding acquisition, Project administration, Resources, Writing – review & editing, Supervision, Validation, Visualization.
